# Supplementary material for: A set of multi-entry identification keys to African frugivorous flies (Diptera, Tephritidae)
Source: Zookeys. 2014 Jul 24;(428):97–108. doi: 10.3897/zookeys.428.7366 (PMC4143993; doi:10.3897/zookeys.428.7366)
Supplement: Supplementary material 4 — Key to Capparimyia [file zookeys-428-097-s004.zip › SF4_ZooKeys_key to Capparimyia/lucid_player/help/help/whskin_info.htm]

WebHelp 5.50 


# WebHelp 5.50 System Info

|  |  |
| --- | --- |
| Start Page | default.htm |
| Skin Name | Lucid3 |
| Generating Time | 17:17 09/24/2009 |
| Language ID | 1033 |
| Compile Script | webhelp5\_compile\_script.xml |
| Compile Build Version | 13.10.606 |
| Product Name | WebHelp 5.50 |
| Authoring Tool Name | RoboHelp X5 |
